# Human retrograde amnesia and memory consolidation

**DOI:** 10.3758/s13423-024-02567-4

**Published:** 2024-09-04

**Authors:** Panayiotis P. Ketonis, Thomas Q. McClelland, Dani Parra, Gabriel A. Radvansky

**Affiliations:** University of Notre Dame, 366 Corbett Family Hall, Notre Dame, IN 46556 USA

**Keywords:** Retrograde amnesia, Memory, Consolidation

## Abstract

This paper reports a reassessment of published literature on the question of whether retrograde amnesia data from patients with severe trauma supports the idea that there is ongoing consolidation of long-lasting memories. That is, memory consolidation continues for decades with older memories being increasingly consolidated, and, thus, more protected from forgetting. Our analysis was limited to patients with specific traumas rather than neurodegenerative conditions that can be complicated by the additional presence of significant anterograde amnesia. These constraints were used because trauma patients have a definitive start to their amnesia allowing comparison of their memories before this event, unlike when there is an undefined amnesia onset. Our results revealed that the standard account of retrograde amnesia only fits part of the data, with more than half not conforming to this account. Specifically, damage to different brain areas was associated with different patterns of retrograde amnesia. Those cases where the standard retrograde amnesia account was held tended to involve damage to the hippocampus and temporal lobes, as expected. Future directions to better understand the influence of retrograde amnesia and memory consolidation are suggested.

## Introduction

The consolidation process protects memories from forces that cause forgetting. As such, it is a valuable tool for studying memory over longer periods. Some evidence for long-term consolidation comes from reports of people who have sustained brain damage resulting in severe retrograde amnesia. These people often have disrupted memories for events closer to the time of the injury, but older memories remain more intact. This points to the effects of a continued process of consolidation on older memories long after their initial encoding. This paper aims to reassess the data on human retrograde amnesia to investigate long-lasting memory consolidation decades into the past.

The use of retrograde amnesia as an indicator of long-lasting memory consolidation stems from Ribot’s (1882) early work on the retention and recovery of information following brain trauma. The landmark finding, known as *Ribot’s gradient*, is that recent memories are most disrupted by physical trauma, with older memories retaining a higher level of preservation. Moreover, as recovery from the trauma progressed, older memories were more likely to return first, followed by recent memories. Newer research has been put forward to support both the idea that newer memories are more likely to be disrupted (e.g., Wixted, 2004, 2005) and that older memories return first (e.g., Roberts et al., 2015). There is clear evidence for shorter periods, in the order of minutes, hours, days, and even weeks (Cahill & Frith, 1995; Lynch &Yarnell, 1973; Squire & Cohen, 1979; Yarnell & Lynch, 1973). Historical data points towards a specific pattern of disruption and preservation that extends even decades into a person's past, which implies that memory consolidation processes continue for decades, resulting in the temporal gradient that we often witness in the results of many experiments (e.g., Dudai, 1996; Eichenbaum, 2001; Haist et al., 2001; McClelland et al., 1995; Meeter & Murre, 2004; Nadel & Moscovitch, 1997).

### Meta-analytic assessment

Given the pervasiveness of the idea that memories continue to undergo consolidation for decades, this area of research would greatly benefit from a meta-analysis. This was done before by Brown (2002), who evaluated studies that assessed long-lasting memories for a range of patients over several decades relative to controls. He compared the performance of amnesia patients with that of control participants by assessing patient performance at different points in time. This was assessed across several different patient types, test types, and so on.

Brown’s key finding was a large performance gap between patients and controls for more recent memories and a smaller gap for older memories. This was true for various causes of retrograde amnesia. From this, he concluded that there is strong evidence supporting the idea that consolidation processes persist for memories formed decades into the past.

This work is appealing because it brought together a wide range of studies and provided a coherent and consistent solution. However, there are some limitations. First, combining the data from trauma patients and patients with neurodegenerative conditions (e.g., Korsakoff’s patients) raises the possibility that the data do not solely reflect the effects of retrograde amnesia, per se, but could instead reflect developing issues with anterograde amnesia, a point noted by Brown (2002). That is, failure to retrieve more recent memories may be due to the memories never having been adequately formed in the first place. In contrast, memory loss in patients with retrograde amnesia caused by specific trauma can be more confidently attributed to the disruption of prior memories.

**Fig. 1:**
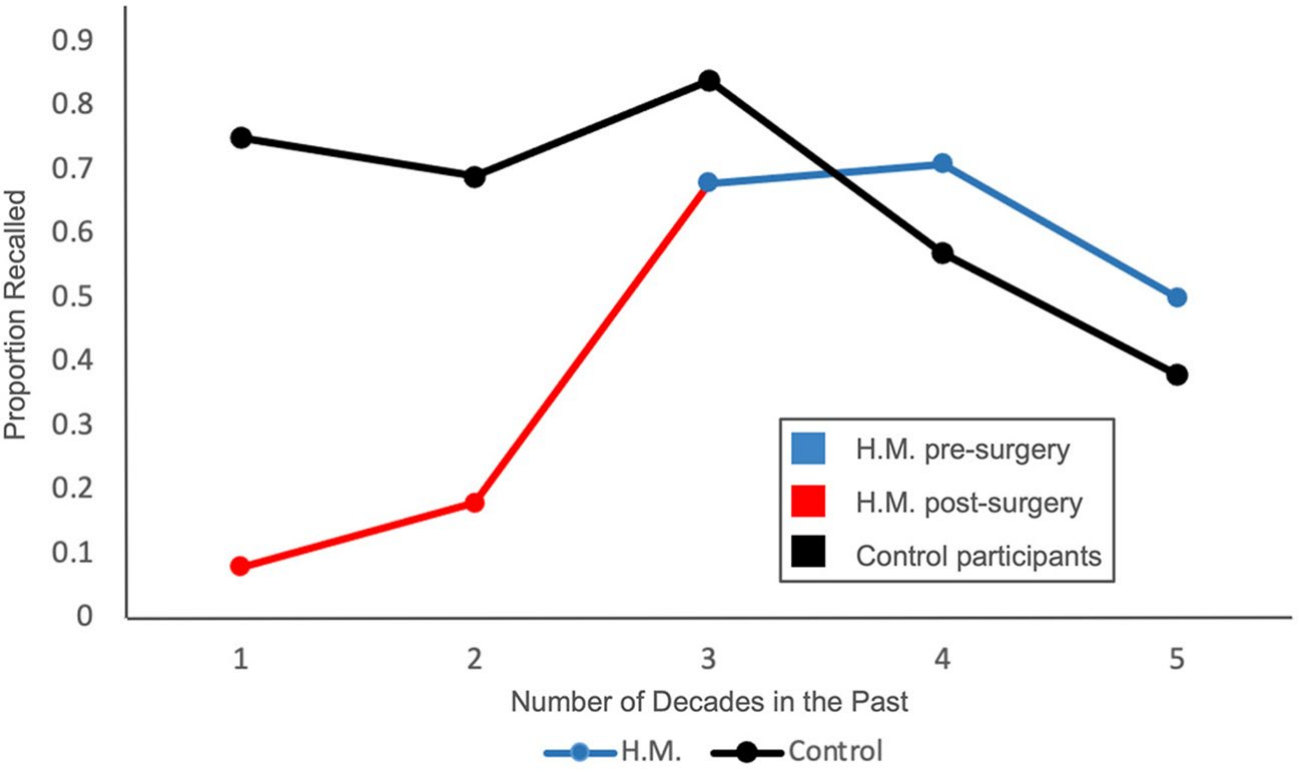
Data from Marslen-Wilson and Teuber (1975) showing the performance of control participants (black line) as well as that of H.M. for memories acquired post-surgery (red) and pre-surgery (blue). In this graph 1 = one decade in the past, 2 = two decades in the past, etc

Thus, the patient data that Brown (2002) included could reflect a combination of anterograde and retrograde amnesia. One example of this is the Marslen-Wilson and Teuber (1975) study of patient H.M. A portion of these data is shown in Fig. 1. The period covered by the study included time both before and after his surgery. Memory ability before the surgery would be affected by retrograde amnesia, and after the surgery would be influenced by his anterograde amnesia. Looking at the data, the story appears to be consistent with the idea that retrograde amnesia is a loss of recent memories: as one progresses further back in time, the memories improve. However, if we only consider the time before surgery (decade 3), there is no evidence of any retrograde amnesia in this data.

Another issue was that the data were considered at the study level with each study being weighed equally; there was no correction for the number of participants. This is problematic because while some studies had multiple patients, others had only one. Thus, small *n* studies may have had a disproportionate influence on the analysis. There were also several cases where the same patients were entered multiple times. This may be because they received multiple tests (e.g., Cermak & O’Connor, 1983), the same data were reported in multiple publications (e.g., Albert et al., 1981; Butters & Albert, 1981), or the same patient was tested at different times (e.g., Reed & Squire, 1998). All these factors complicate the assessment of performance.^1^

Here, we aim to address these issues in the current analysis and expand upon this assessment by also including any new data that we have been able to garner from the literature. We assessed change in memory performance for patients and controls separately, using only patients in which there was a definitive onset to the retrograde amnesia and no influence from anterograde amnesia. We then weighted the data according to the number of participants.

### Criteria for Inclusion

Several criteria were used for our reassessment of the retrograde amnesia data. The first was that we limited ourselves to cases in which there was a clear single onset of retrograde amnesia. This avoids confounding effects from anterograde amnesia, in which the original encoding of the information is disrupted. It also avoids cases in which memory deterioration occurs over time in an unpredictable way (e.g., neurodegenerative disease).

Second, for a period of time to be considered in our analyses, it could not overlap with any time in which there was clear, identifiable anterograde amnesia. For example, the most recent two decades of the H.M. data would be dropped from the analyses because it was during a time when dense anterograde amnesia was present. Thus, this poorer memory can be attributed to the poorer encoding of the memories at the outset, not to retrograde memory loss.

A third criterion is that there be three or more retention intervals. When there are only two, only linear retention functions are possible. This is problematic because many memory functions have been captured by power, logarithmic, and other functions (e.g., Rubin & Wenzel, 1996). These functions cannot be modeled using only two retention intervals.

A fourth and final criterion involves using the most recent data available from patients who were assessed multiple times. This avoids assessing patients when they are still recovering from their trauma, thereby providing a clearer assessment of how memory was affected by retrograde amnesia.

### Patterns of data

For Brown (2002), the assessment of consolidation over decades was done by dividing the patients’ memory score by the control group’s memory score. Thus, a greater degree of similarity between patients and controls equates to a higher percentage. This was done with the idea that if Brown’s hypothesis was correct, older memories would show smaller and smaller discrepancies in performance between patients and the control group. Ultimately, what was important for Brown’s assessment was the increase in percentage overlap between the two groups as the age of the memory increased (i.e., with each decade).

Brown’s (2002) assessment revealed that across the decades there was an increase from 2 to 11 percentage points per decade as the age of memory increased (*M* = 8.1) (his Fig. 1), with patient performance increasing from around 48% of the controls in the first decade, to about 73% of the controls by the fourth or fifth decade prior to testing. Thus, for older memories, performance is less and less disrupted compared to that of the controls. Moreover, this increase was largely linear across the decades.

While this approach seems sensible at first glance, it does lose some qualitative aspects of the data. Specifically, there could be cases in which there is a smaller and smaller difference between the two groups, but not because the patients are improving to the level of the controls. More specifically, it fails to differentiate between the patients improving and the controls deteriorating.

**Fig. 2:**
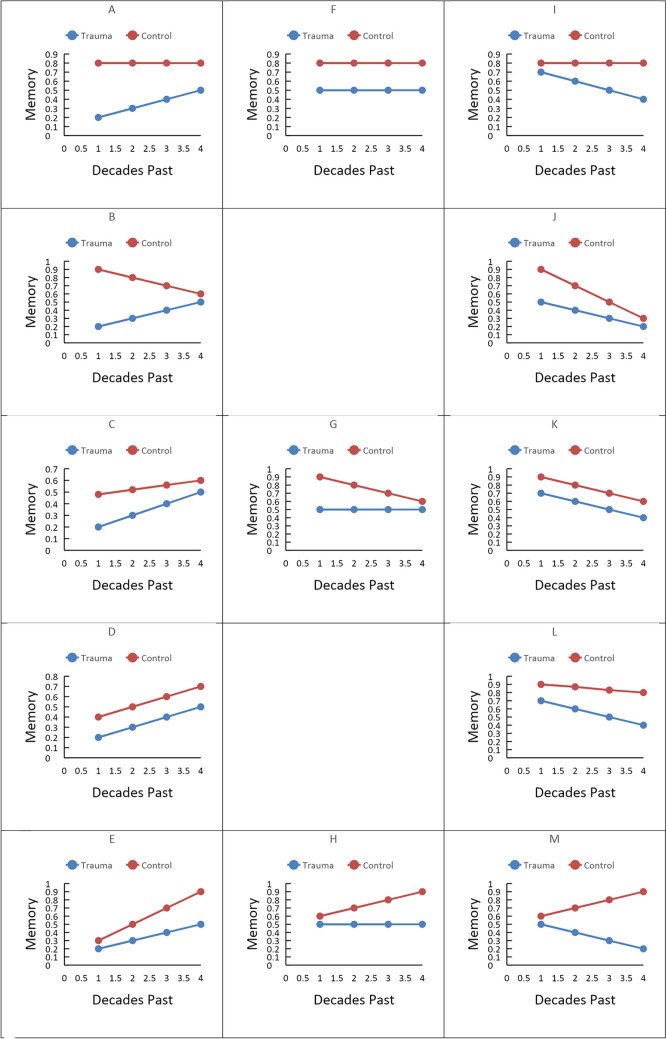
Possible patterns of data over time for trauma patients and control participants

To illustrate, a range of possible patterns of performance are shown in Fig. 2. The patterns most consistent with a view in which consolidation persists over decades are patterns A, B, and C. In all three of these cases, patient performance is improving to be more in line with the controls. Theoretically, this could be due to consolidation strengthening the patients’ older memories, similar to the memories of the controls. However, some patterns also produced reduced differences between patient and control participant performance, but do not support this standard model of consolidation. This can be seen in pattern G (in which there is no change in patient performance, but control performance grows worse), and pattern J (in which both patient and control performance is declining over time, but the control performance is declining faster). Pattern G seems to show that for both trauma and control patients, consolidation, at best, only works to preserve older memories but not improve their retrieval. These differences were not assessed by Brown (2002), nor was the relative occurrence of these various patterns reported.

### Analyses

The data were analyzed in three ways. The first concerned the *decade-by-decade data* in which memory was probed for information coming from a variety of decades prior to the onset of retrograde amnesia. The second focused on *lifetime periods data* in which autobiographical memory was originally assessed in terms of whether it was in one of three time periods, namely: recent, early adulthood, or childhood. These two different analyses are separated because of the different ways that these two types of memories have been reported in the literature. The third was an assessment of which brain areas are associated with the different patterns of performance.

#### Decade-by-decade analysis

The data that met our criteria is presented in the Online Supplementary Material (https://osf.io/526pz/). Note that for studies in which there was clear evidence of anterograde amnesia decades prior to the assessment (Cermak & O'Connor, 1983; Marslen-Wilson & Teuber, 1975; Parkin & Hunkin, 1991), the data from those decades were dropped.

We first considered the rate at which the patterns of data could be classified into the forms in Fig. 2. We made this classification using the average proportion change from one decade to the next. When this average proportion change was .03 or less, we classified this as a flat pattern of performance. When both the trauma patient and control data increased or decreased, they were classified as “different” if one demonstrated a > .03 increase or decrease compared to the other. Rather than analyzing this on a study-by-study basis, as was done by Brown (2002), we reported weighted means based on the number of participants.

Moreover, because we considered the type of information that was tested (e.g., famous faces) and the memory measure (i.e., recall or recognition) to provide more sensible assessments, we further trimmed the data. Specifically, we removed any cases in which there was incomplete data for the first two decades. This was done because of the presence of dense anterograde amnesia (Cermak & O'Connor, 1983; Marslen-Wilson & Teuber, 1975; Parkin & Hunkin, 1991). If the standard account is correct, then most of the memory loss would be for these decades

**Fig. 3:**
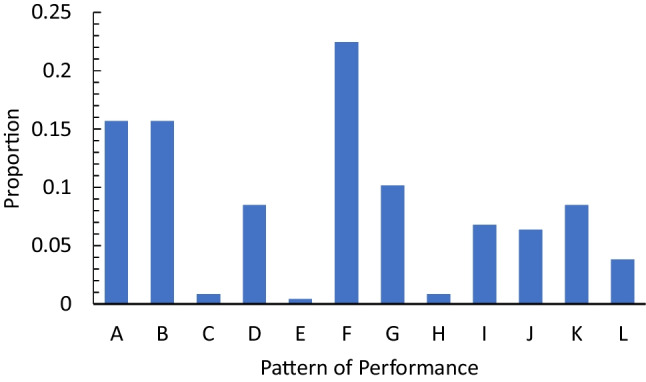
Distribution of patterns of data for the complete data set adjusted for the number of trauma patients

We also removed data from those studies in which, for a given measure, there was only a single study that contributed to a retention interval. This cut included the sixth-decade data from the Kapur et al. (1986) study (which was also removed by Brown (2002)), the fourth-decade data for the Costello et al. (1998) study, the fifth-decade of the Squire et al. (1990), and the fourth and fifth decades of the Salmon et al. (1988) study. Using this approach, the distribution of data into the various response pattern categories is shown in Fig. 3.

As can be seen, while some data conformed to the patterns consistent with the standard account of retrograde amnesia (patterns, A, B, and C), the majority (68%) did not. Thus, Ribot’s gradient as a descriptor of memory performance after cortical damage is, at best, incomplete. To address why this might be the case for the decade-by-decade analysis, we assessed whether the observed patterns are due to the types of materials or memory test used.

#### Material and task types

Across these studies, different materials and memory task types were used. The materials could be visual or auditory, and the tests were either recall or recognition tests. Before looking at the pattern of performance for these, we provide a brief description of each.

For *Famous Faces Recall and Recognition*, people were given photographs of people who became famous during a particular decade, and the task was either to name those people or to indicate whether the name provided was the name of that person. For *Public Events Recall and Recognition***,** people were given descriptions of public events that occurred during a decade. These were presented as questions (e.g., Who killed John Lennon?) and people were either asked to recall the information pertaining to that event or to indicate which of several options corresponded to the answer. For *Dead or Alive Recognition*, people were given the names of well-known personalities with the task of indicating who was alive and who had died. For *Famous Voices Recall and Recognition,* people heard famous people speaking (several from each of multiple decades) and were to either recall the names of those people or select who the speaker was from a list of choices. Note that these tasks were only reported in a single study (Kapur et al., 1986), with a single trauma patient and five controls. For *New Vocabulary Recall and Recognition,* people were given words that had come into use during a specific decade and asked to either recall their meanings or to indicate which of several options was the actual meaning. This task was only reported in a single study (Reed & Squire, 1998), with two trauma patients and four controls. For *Famous Names Recall and Recognition*, people were either given partial names of people who were famous during a particular decade and asked to complete the rest of the name (e.g., Alfred Hitch_____) or given the names of famous and non-famous people from a particular decade and tasked with indicating which names were famous. For *Events and Names Recall and Recognition,* studies used both the *Famous Faces* and *Public Events Recall and Recognition* tests and collapsed the data. Note that the recognition data for this task type reflect only a single study (Salmon et al., 1988) with one trauma patient and 14 controls. For *Television Show Recall and Recognition*, people were given photos from television shows that were broadcast during a particular decade and asked either to recall the names of those shows or recognize which of four names corresponded to the names of shows. Note again that these data reflect only a single study (Tanaka et al., 1999) with one patient and one control participant.

#### Material and task performance

**Fig. 4:**
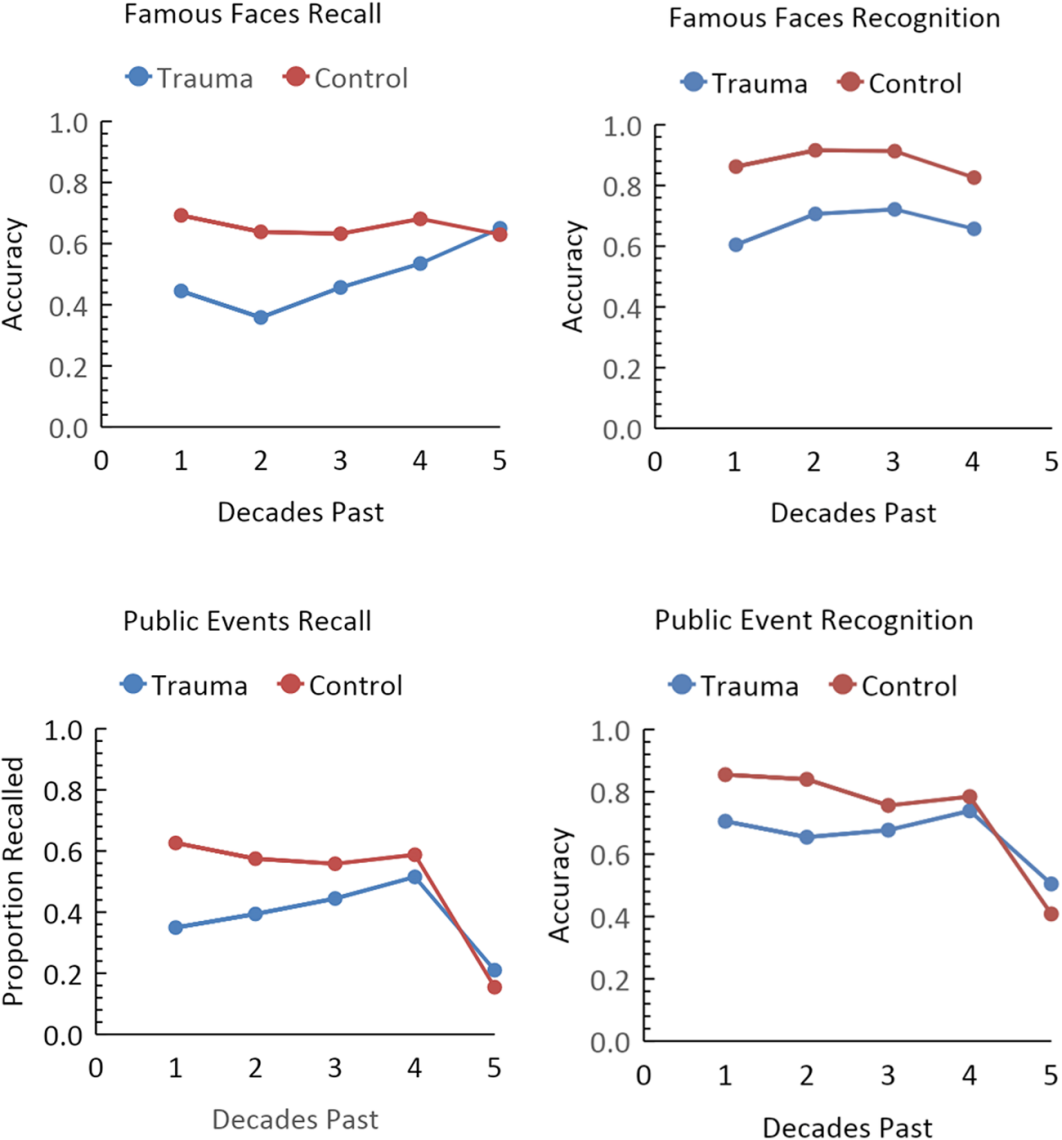
Performance of trauma patients and controls adjusted for the number of participants

To assess performance by material types and task combinations, we averaged performance for trauma patients and controls across studies and adjusted for the number of participants for each of these material types and tasks (Fig. 4). Only the tasks involving famous faces and public events are presented here because of the very small number of observations for the other tasks. These other material types and tasks are included in the online supplementary material (https://osf.io/526pz/). Table 1 presents the average change per decade for the trauma patients and controls for each of the measures, as well as Brown’s (2002) characterization of the percent change of patients relative to controls.

**Table 1:** Change over time for trauma patients and controls for each measure

Measure	Avg. change for patients	Avg. change for controls	Net change	Pattern type	Brown (2002) percent change
*Decade-by-decade analysis*					
Famous Faces Recall	.05	-.02	.03	A	10
Famous Faces Recognition	.02	-.01	.01	F	3
Public Events Recall	-.03 (.06)	-.12 (-.01)	.09 (.05)	G (A)	20 (11)
Public Events Recognition	-.05 (.06)	-.11 (-.01)	.06 (.05)	L (A)	10 (11)
*Time periods autobiographical memory analysis*					
Incidents	.03	-.01	.02	F	4
Personal Semantic	.06	-.01	.05	F	7

The information in parentheses are the findings when extreme values from the oldest memories are removed

Looking across the decade-by-decade analyses, there was some variability in terms of whether the data fit the standard account. There does not appear to be a clearly predictive factor in terms of the task type that can identify when the standard account will be observed. The data are more or less evenly split between recall and recognition tests. Similarly, there does not seem to be any particular material type that leads to one type of retention pattern over another. Thus, the variation in patterns of performance for patients and controls for these decade-by-decade analyses does not appear to be a result of either of these factors.

#### Forgetting curves

As mentioned earlier, one of our criteria for selecting data to include in our analyses is that there be at least three retention intervals. This is because when fitting forgetting curves (such as a power function), then at least three retention intervals are needed. For this assessment, we fit two functions to the data. The first was a power function. This was done because many studies have suggested that power functions fit a wide range of data (e.g., Wixted & Ebbesen, 1991). The other was a linear function. This was done because more recent work has found that some data are better fit by a linear function (Fisher & Radvansky, 2019.). This is important because a power function exhibits a constant proportion loss over log time, while linear functions show an increasing proportion loss over log time. This suggests that very different qualitative factors are at work. Moreover, it appears that power functions are more likely to be observed when information has been learned to a lesser degree, whereas linear functions are more likely to be observed for information that has been better learned (Fisher & Radvansky, 2022). It is possible that patients and controls differ in the pattern of retention and forgetting, which could reflect further qualitative differences in the memory representations or processes for older knowledge.

To this end, we found the best fitting power and linear functions for the patient and control participant data for each of the memory tasks used in our data set. The *r*^*2*^ values for these best fitting functions are reported in Table 2.

**Table 2:** Regression analysis for trauma patients and controls for each measure

Measure	Function type	Patients	Controls
Famous People Recall	Power	.360	.167
	Linear	.545	.208
Famous People Recognition	Power	.637	.555
	Linear	.831	.688
Public Events Recall	Power	.320	.356
	Linear	.443	.630
Public Events Recognition	Power	.374	.492
	Linear	.606	.781
Dead or Alive Recognition	Power	.471	.279
	Linear	.633	.472
Famous Names Recall	Power	.093	.015
	Linear	.226	.002
Famous Names Recognition	Power	.178	.006
	Linear	.141	.000

In most cases, the data were better fit by a linear than a power function. The only exceptions were “famous name recall” for controls and “famous name recognition” for both groups. It is also important to note that there were very few observations for these data, and many of the fits are very poor to begin with. That said, when taking all measures into account, the superior fit of a linear function is consistent with the idea that the information was well learned. This is not surprising given that the tasks are tapping knowledge that was retained over the course of decades.

The second thing to notice is that similar patterns were observed for the patients and controls. This suggests that the basic memory representations and encoding processes involved for the two groups are likely similar. What differs is the effectiveness of the retention and retrieval of memories.

## Lifetime periods autobiographical memory analysis

For this analysis, we used studies in which people were asked to provide autobiographical memories from different periods of their lives. In these publications, these periods were referred to as recent, early adulthood, and childhood. One issue with these data is that it is harder to say when the amnesia-inducing trauma occurred relative to the items probed. Specifically, it is unclear whether items probed for the recent time period were available before or after the onset of the amnesia. Thus, these results need to be considered with this qualification in mind.

**Fig. 5:**
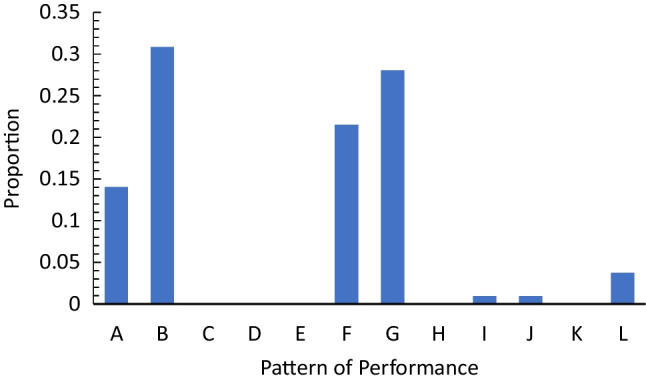
Distribution of patterns of data for the complete autobiographical data set adjusted for the number of patients

We first sorted the data into the same patterns presented in Fig. 2 based on the proportion of memories given for a certain lifetime period. The resulting distribution of patterns is shown in Fig. 5. As shown, approximately 55% of data did not conform to the standard account (patterns A, B, and C). Thus, again, there is a substantial amount of the data that is not captured by the standard account of retrograde amnesia.

There were two types of tasks for the autobiographical memory assessments. For *autobiographical incidents*, people were asked to recall specific autobiographical events in response to a cue, such as recalling a memory involving a certain teacher. In comparison, for *personal semantic information,* people were asked to report semantic information such as the names of the schools attended. Performance for each of these task types is considered in turn.

### Incidents

**Fig. 6:**
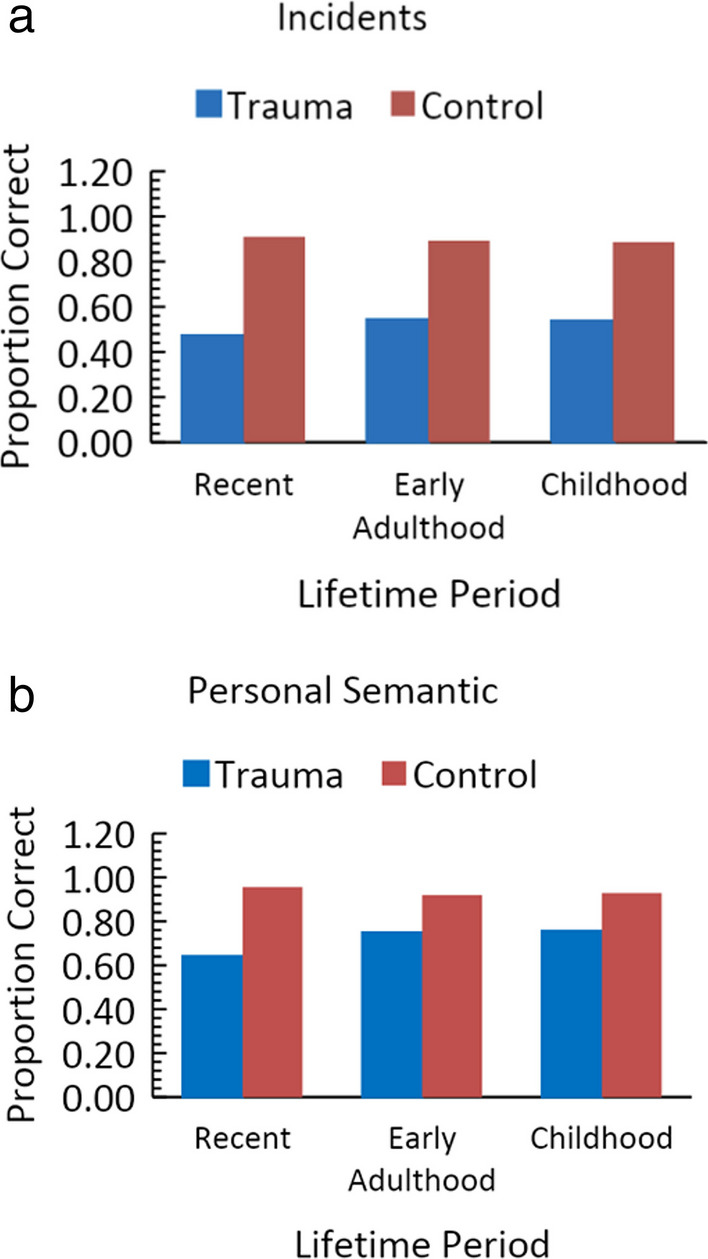
**a** Performance of trauma patients and controls, adjusted for the number of participants, for the autobiographical incidents. **b** Performance of trauma patients and controls, adjusted for the number of participants, for the personal semantic memory tests

Performance for trauma patients and controls averaged across studies and adjusted for the number of participants is shown in Fig. 6a. As can be seen, performance did not follow the standard account of retrograde amnesia and consolidation, but instead is best fit by pattern F. This pattern demonstrates small or no average changes.

### Personal semantic

Performance for trauma patients and controls is shown in Fig. 6b. The patient change was limited to the transition from recent information to young adulthood information. Performance on information from a patient’s childhood showed no change from early adulthood. Given this limitation, the task also fails to produce data consistent with the standard account of retrograde amnesia.

### Brain area assessment

Thus far, we have considered the data patterns for a variety of tests. This has shown that the standard account of retrograde amnesia is not as reproducible as many might think. That said, it is important to note that this analysis included patients with a wide range of pathologies, with different parts of the brain being affected. It may be that damage to some brain areas is more likely to produce the standard pattern compared to others. To this end, for each of the studies that conform to each pattern, we detailed which parts of the brain were affected.

The online supplementary material includes a tabulation of the studies assessed here, the patterns that are fit by the data, the type of memory task involved, and the cause of the damage. For this analysis, *frontal* includes damage to the ventral frontal cortex, orbital frontal cortex, left frontal lobe, and right frontal lobe. The *hippocampal complex* refers to damage to the hippocampus, fornix, amygdala, parahippocampal gyrus, and the uncus. *Temporal* refers to damage to the lateral temporal cortex, as well as either the left or right temporal lobes. *Ventricles* refers to damage to the right and left ventricle areas. Finally, *thalamic* refers to damage to the thalamus, anterior thalamus, and mammillary bodies.

**Fig. 7:**
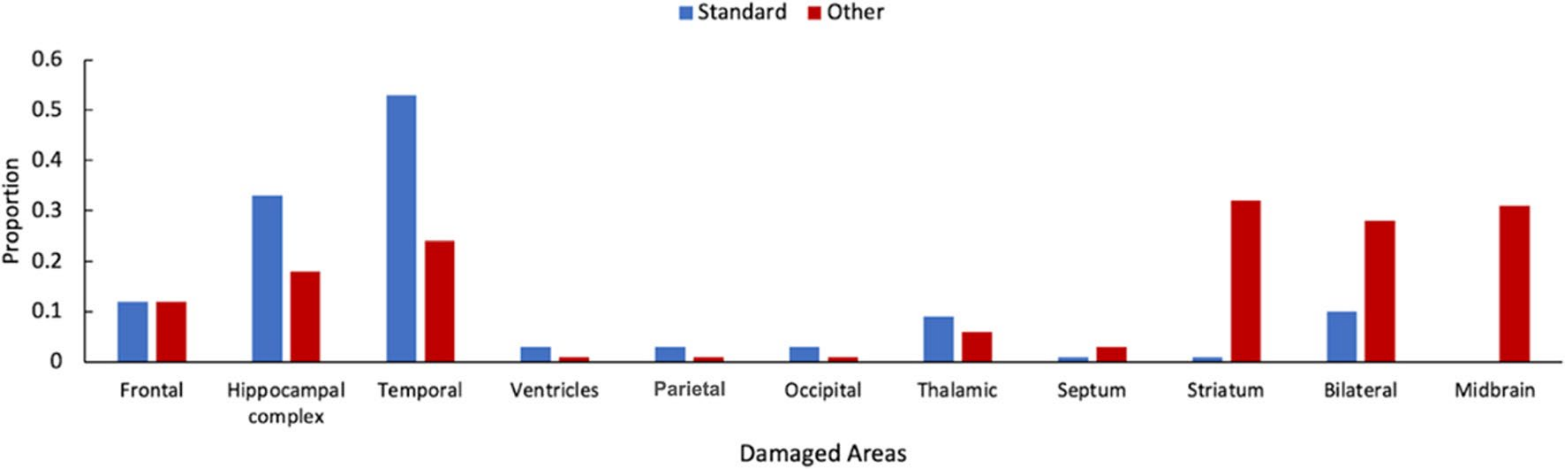
For the decade-by-decade tests, the location of damage for patterns of performance either conformed to the standard account of retrograde amnesia or conformed to some other pattern of performance

A comparison between the damaged areas and the account of retrograde amnesia present in the decade-by-decade analysis can be seen in Fig. 7. Patterns that conform to the standard account of retrograde amnesia are more likely to involve damage to the temporal lobes or the hippocampal complex. In comparison, patterns which do not conform to the standard account are more likely to be observed when the damage is in the midbrain, striatum, or was simply described as bilateral damage.

These findings suggest that not only can retrograde amnesia result from damage to different brain areas, but also that the specific brain areas damaged can influence the resultant amnesia. In other words, there appear to be different types of retrograde amnesia. The classic Ribot’s gradient is more likely to be observed when areas traditionally associated with declarative memory are damaged. In comparison, other patterns of memory performance are more strongly associated with damage in other areas. This is likely because damage to these areas is not disrupting memory consolidation or retention per se, but is likely influencing factors involved in the retrieval, assessment, and reporting of memories.

**Fig. 8:**
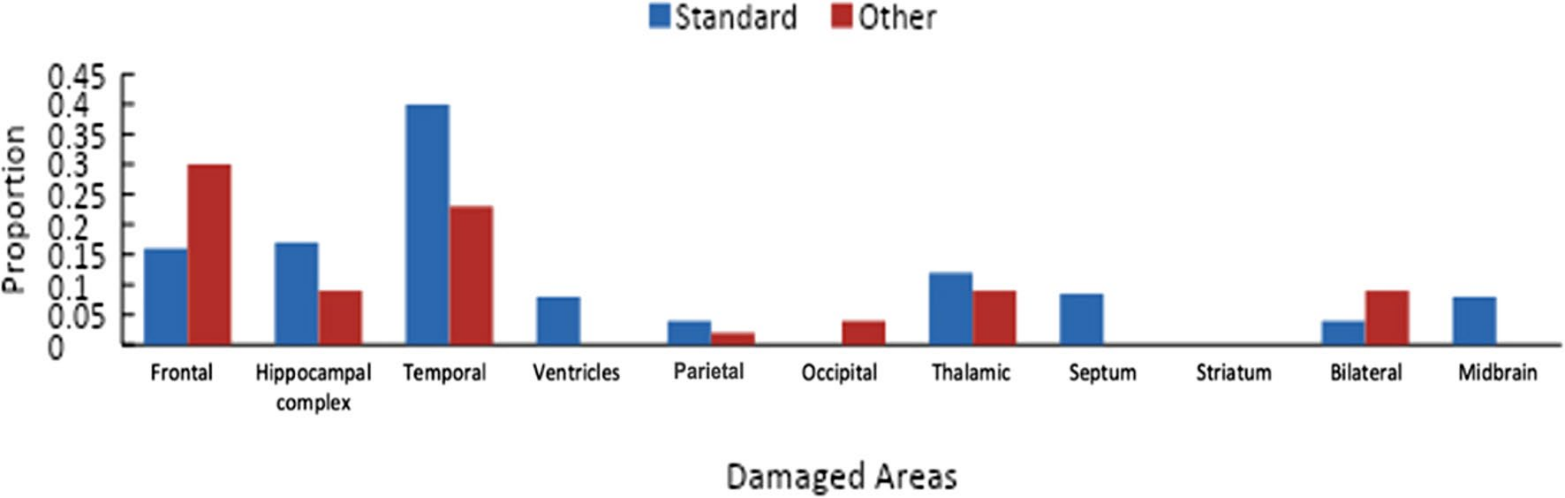
For the autobiographical memory tests, the location of damage for patterns of performance either conformed to the standard account of retrograde amnesia or some other pattern of performance

A similar analysis was done for the autobiographical memory tasks, and the results are shown in Fig. 8. As can be seen, patterns that conform to the standard account of retrograde amnesia are more likely to involve damage to the temporal lobes or the hippocampal complex.

### Brown-style analysis

In his paper, Brown (2002) compared memory ability of patients and controls by measuring the gap in performance on different memory tasks. If the size of the gap decreased, this was interpreted as indicating improving performance by patients relative to controls. If the size of the gap increased, it indicated the opposite. The aim of this section is to see how our data set would do when analyzed in this way.

To this end, the data assessed here were analyzed using the same approach. At each datapoint included in each individual memory task, the difference between control and patient performance was calculated. Using those points, the slope was taken for each study. Then, the weighted average of these slopes was calculated for each unique memory task type. For example, all slopes from the famous faces recall task were weighted for the number of participants in each individual study, then averaged to yield a total slope of -0.05 for that task. In other words, the performance gap between patients and controls in a famous faces recall task decreased by 5% over five decades of memory testing. A summary of the findings from this analysis can be seen in Table 3. Using this approach, the data from our sample of studies are in line with that reported by Brown.

**Table 3:** Percent change of the performance gap between patients and controls for each unique task type. Each value is a weighted average of all studies including the specified memory task. A negative value indicates a decreasing gap as older memories are tested

Task type:	Change in performance gap:
Famous faces, recall	-5%
Famous faces, recognition	-5%
Public events, recall	-6%
Public events, recognition	2%
Dead or alive, recognition	-10%
Famous names, recall	-7%
Famous names, recognition	-6%
Events and names, recognition	-10%
Events and names, recall	-10%

## General discussion

A prominent issue for research on memory is the fate of memories over long periods of time. A dominant view is that memories consolidate and become more permanent and resistant to forgetting over the course of years. One salient source of support for this idea is that when there is retrograde amnesia, the disruption is more prominent for more recent memories, and older memories tend to return before newer memories. This is Ribot’s (1882) gradient. The aim of this project was to assess this idea by building off a meta-analysis done by Brown (2002) that was interpreted as providing evidence for a general years-long consolidation process. However, this approach included data contaminated by other sources of memory loss beyond retrograde amnesia, did not adjust for the number of participants, and did not consider the patterns of loss for patients and controls.

The results of our re-analysis suggest that the support for prior conclusions about retrograde amnesia and consolidation has been overstated. The traditional view of retrograde amnesia following an injury event is that recent memories will be disrupted to a greater extent than older memories. Older memories have existed for longer – therefore, they have had more time to consolidate. Thus, the standard view of retrograde amnesia states that memory shows Ribot’s gradient of accessibility.

While many data sets do conform to this pattern, there were also many reports that we reviewed that did not. For those cases that did not conform, the differences in performance between amnesiacs and controls were either stable across different retention intervals or the amnesiacs diverged from the controls. Thus, damage to memory resulting in retrograde amnesia produces a range of different patterns of data. This was true both for the decade-by-decade analysis and the autobiographical data.

There were no noticeable influences of material and task types in terms of whether the data conformed to the standard view or some other pattern. However, there were some notable patterns in the type of brain damage incurred and the pattern of retention and forgetting. Specifically, damage to the temporal lobes or to the hippocampal complex (neurobiological structures strongly associated with declarative memory) increases the likelihood of standard patterns.

However, retrograde amnesia takes a very different form when the damage is localized in other structures, such as the striatum, thalamus, or midbrain. Two of the most common trends that emerged from damage to these areas were pattern F and pattern G. In pattern F (as well as D), there is no change in patient performance relative to control performance – the slope of both lines either remain flat or descend at the same rate. The size of the gap between patient and control is maintained. In pattern G, there is no change in patient performance, but control performance worsens over time. These patterns of performance suggest some mechanism other than a disruption of some consolidation process. For pattern F, and likely D, this indicates a problem with retrieval, not consolidation, because the deficit is relatively consistent across different delays. For pattern G, this is harder to pin down, but could also be a retrieval problem, with older memories, which may require more reconstruction at retrieval and such, having less information retrieved.

The idea that some aspects of retrograde amnesia may be due to retrieval deficits rather than consolidation is not new. Gisquet-Verriera and Riccio (2018) suggested that many aspects of memory retention and loss that have been attributed to consolidation, such as a failure to retrieve because of encoding specificity problems, could be accounted for by processes of memory integration and retrieval processes. Here, we do not argue for a whole-sale disposal of the idea that some retrograde amnesia is caused by a disruption of consolidation processes but instead that some types of severe memory loss can be due to the disruption of other memory processes, such as retrieval along the lines suggested by researchers such as Gisquet-Verriera and Riccio.

Consistent with the idea that these patterns reflect retrieval, rather than consolidation, problems, neuroimaging and neuropsychological evidence suggest that there is significant striatum involvement in declarative memory retrieval (Scimeca & Badre, 2012). Thus, striatal lesions could lead to retrieval issues that present in our data as memory loss. This is in line with the brain area assessment that revealed that striatum damage often led to non-standard patterns of amnesia.

The involvement of the thalamus in the production of retrograde amnesia is more ambiguous. Damage to the thalamus results in patterns of retrograde amnesia that both does and does not conform to the standard consolidation account, and to equal degrees. The thalamus has also been implicated in both long-term memory encoding and retrieval (e.g., Pergola et al., 2013), although that involvement is not clear (Geier et al., 2020). The involvement of midbrain structures is also ambiguous. While midbrain damage is associated with nonstandard patterns of retrograde amnesia for the decade-by-decade analyses, the opposite was true for the autobiographical memory assessments. The midbrain, and its relationship to structures such as the hippocampus, is known to be involved in dopaminergic processes related to memory encoding (e.g., Schott et al., 2004; Shohamy & Wagner, 2008). However, the involvement of the midbrain in memory retrieval is not well known, and the literature on its involvement in retrieval is thin (e.g., Clos et al., 2019).

When the data were analyzed using techniques similar to Brown (2002), it showed the same overall pattern as was reported in that paper. In eight of the nine distinct memory tests, the performance gap between patients and controls decreased as the age of the memory increased. Specifically, the largest decreases in performance gaps took place in three types of tests: event and name recognition tests, television show recognition tests, and dead/alive recognition tests. All three of these analyses yielded 10% decreases in performance gaps. The one testing type that showed an increased performance gap was the public event recognition task, which yielded a relatively small 2% increase, which is essentially no change. The remaining five tests showed decreases in performance gaps between 5% and 7%. Of note, when re-analyzing our paper using Brown’s methodology, the number of participants in each study was considered, and weighted accordingly, which was not done by Brown. Overall, this suggests that, although we used a different subset of the data, it did not differ meaningfully from that used by Brown, at least in terms of the way that he assessed the influence of amnesia over time.

The finding that most of the forgetting loss patterns were best fit by a linear function compared to a power function is telling. This was observed for both the patient and control data across the memory task types. While the general assumption is that memory loss follows an Ebbinghaus-like pattern (with greater forgetting earlier on and less as time goes on, as with a power function), more recent work suggests that linear patterns of performance can be observed (Fisher & Radvansky, 2019). Specifically, linear patterns of loss are more likely to be observed with well-learned knowledge. Thus, the types of knowledge that are assessed in these studies was often well-learned to begin with.

This brings us to the issue of just what kind of information is being probed for in these studies. For the decade-by-decade studies, much of the information was likely repeated to the person multiple times, such as famous events or faces. Thus, this knowledge is more likely to have a semantic quality, and is much less episodic, even if those initial exposures were more likely to be confined to a particular period of time. They are not the episodic or autobiographical event memories that are often tested when researchers are testing memory retention and forgetting over time. This needs to be kept in mind when evaluating this kind of data. This is less of an issue for the studies that use autobiographical memory tests. However, the problem there is that it is harder to pin down the age of the memories, know their veracity, and be able to look at patterns of performance over time (e.g., fit forgetting functions).

Another issue that was raised by one of our reviewers is that many of the memory tests used in these studies are curated tests. This was likely done to avoid floor and ceiling effects. That is, items were selected from each time period to keep performance off the floor for patients and the ceiling for the controls. Moreover, it may be that more memorable older events may be selected to achieve similar levels of performance relative to recent events. Thus, the retention and forgetting curves may not be as dramatic as might otherwise be the case.^2^ That said, even if there is some element of bias to the creation of the tests, our primary concern was with differences between patients and controls, and so, while such a practice might attenuate the degree of a forgetting curve, it may not be a major player in our assessment here.

Overall, going forward, these results suggest that further work is needed to understand the mechanisms that can result in retrograde amnesia. While the role of the medial-temporal complex is better understood, as well as its relation to a typical Ribot’s gradient of loss, the involvement of other brain areas is less well understood. Given that damage to these areas is more likely to result in a pattern of loss that is more consistent with memory retrieval difficulties, a better understanding in how they are involved in these memory processes can also aid our understanding of the cognitive mechanisms that are at work during memory retrieval. Moreover, these results could also be used to help the diagnosis of a traumatic brain injury. Damage to different brain areas produces different patterns of retrograde amnesia. This should be kept in mind when administering memory tests to new patients. Examining and comparing the pattern of memory loss on patients' memory tests to the patterns shown above, could be another diagnostic tool in differentiating between neurodegenerative conditions.

## Data Availability

The datasets used for the current study are available via the Open Science Framework at https://osf.io/526pz/.

## Footnotes

1 On a more minor note, in a few cases, Brown (2002) reported using data that was different from the data sets he claimed to (e.g., Squire & Slater, 1978), and data reported in the manuscript that did not match the data in the published work (e.g., Costello et al., 1998). That said, this likely had only a modest influence on the outcome.

2 The television test may be one exception to this, because single season shows are set by what actually happened in the world, not a test creator’s possibly subjective selection criteria.
